# Human detection thresholds of DC, AC, and hybrid electric fields: a double-blind study

**DOI:** 10.1186/s12940-021-00781-4

**Published:** 2021-08-21

**Authors:** Michael Kursawe, Dominik Stunder, Thomas Krampert, Andrea Kaifie, Sarah Drießen, Thomas Kraus, Kathrin Jankowiak

**Affiliations:** 1 Research Center for Bioelectromagnetic Interaction (femu), Institute for Occupational, Social and Environmental Medicine, Uniklinik RWTH Aachen University, Pauwelsstraße 30, 52074 Aachen, Germany; 2 Institute for High Voltage Equipment and Grids, Digitalization and Power Economics, RWTH Aachen University, Aachen, Germany; 3 Institute for Occupational, Social and Environmental Medicine, Uniklinik RWTH Aachen University, Aachen, Germany

**Keywords:** Exposure, Signal detection theory, Perception, Psychophysics, Energy transition, Energy transmission

## Abstract

**Background:**

In the course of the ongoing transition of electric energy systems, transmission corridors are often upgraded to higher voltages and other technologies leading to another quality of human exposure. The study aims to determine human detection thresholds for direct current (DC), alternating current (AC), and hybrid electric fields (various DC; constant AC).

**Methods:**

A total of 203 participants were exposed to DC, AC, and hybrid electric fields (EFs) in a highly specialized whole-body exposure laboratory using a double-blind experimental setting. Additionally, the participants were exposed to ion currents in part of the DC and hybrid sessions. To investigate environmental influences, relative humidity was changed in two subgroups during EF perception. Methods derived from the signal detection theory and the adaptive staircase procedure based on the single interval adjustment matrix were used to assess individual sensitivity and detection thresholds, respectively.

**Results:**

The results indicated that detection thresholds of hybrid EF were lower compared to single EF presentation of DC or AC. Ion current exposure enhanced EF perception. High relative humidity facilitated DC EF perception, whereas low relative humidity reinforced the perception of AC EFs.

**Conclusions:**

With this systematic investigation of human perception of DC, AC, and hybrid EFs, detection thresholds were provided, which can help improve the construction processes of energy transmission systems and the prevention of unwanted sensory perception by contributing to the determination of limit values.

## Background

As a central component of modern society, electric energy is required in every enterprise or household and is becoming increasingly important in fuel-free transportation. Today, most electric energy is produced in power plants or wind farms far apart, and transportation to the user is often achieved via high-voltage overhead lines. Worldwide, energy transmission is realized mostly with high-voltage alternating current (HVAC) that has a good efficiency for short and medium distances. Over long distances, high-voltage direct current (HVDC) transmission is low-loss and thus more cost-efficient [1]. HVDC overhead lines already exist in several countries, such as China, Canada, or the United States, whereas, in Germany, this technique is currently in planning. Despite this development, no limit values for DC electric fields (EFs) are provided due to the lack of data. Furthermore, overhead power lines can produce corona ions due to electrical discharge [2]. Because HVDC overhead lines will exist more and more next to residential areas, the interaction with humans becomes more important, particularly the aspect of how EFs and corona ions within its corridor can produce a conscious perception, for instance, when going out for a walk or are at work next to it. This becomes even more interesting when both HVAC and HVDC overhead lines are mounted in close vicinity leading to hybrid EF.

### EF perception

Despite early articles on AC EF highlighted the need research on the interaction of humans and EFs [3, 4], only a few experimental studies on human EF perception exist showing rather heterogeneous results in terms of just perceptible EF strengths (for review of static EF, see [5]; for review of AC EF, see [6]). Chapman et al. [7] exposed the participants’ hand and forearm, whereby nobody was able to perceive DC EF of up to 65 kV/m. In contrast, nearly half of the participants were sensitive to AC EF of up to 35 kV/m, although the interindividual variance was large. In another study, the human detection threshold of DC EF during forearm exposure was about 375 kV/m [8]. An increased relative humidity to 90% led to a 30% decrease in detection thresholds. Moreover, both research groups stated that body hair is the most essential factor facilitating EF perception [7, 8]. This was supported by the finding that relative humidity of scalp hair influenced EF perception [9]. Nevertheless, the general sensitivity of the body surface toward vibrotactile stimuli was not investigated in this context. Interestingly, detection thresholds under whole-body exposure were lower [10, 11]. Clairmont et al. [10] placed volunteers below a hybrid test line at different measurement points and recorded ratings of field perception. In this nonblinded investigation, the DC EF detection threshold was estimated at about 20 kV/m, whereas, under the hybrid condition, when the AC EF strength was 10 kV/m, a DC EF strength of 5 kV/m was just perceptible [10]. In a well-designed experimental study, Blondin et al. [11] used a specialized exposure facility [12] to investigate human DC EF perception under whole-body exposure and the additional influence of corona ions on perception. On average, the participants could detect a DC EF strength of 45.1 kV/m that decreased to 36.9 kV/m when an ion current density of 120 nA/m^2^ was presented. Whereas one-third of the participants could detect EF strengths of 25 kV/m, another third was not able to detect the maximum EF of 50 kV/m. When asked, the participants described their sensation mainly related to hair movements at the scalp, face, or arms [11]. These perception mechanisms related to hair movements were described as well in terms of AC EF perception [6]. Regarding factors influencing EF perception, Reilly [6] delineated low temperature and relative humidity to lower AC EF perception ability.

In a recent study of our group, Jankowiak et al. [13] outlined experimental and environmental factors influencing EF perception. Therefore, a highly sophisticated whole-body exposure laboratory was built based on the study of Nguyen and Maruvada [12], where DC EF with and without ion current, AC EF, and hybrid EF could be generated. Eleven participants, each over five test days, were examined, and individual detection thresholds were calculated. Variations in experimental factors, such as exposure duration, ramp slope (onset time of the EF), and the presence of ions, and the environmental factor relative humidity were investigated. The average detection thresholds were 23.4 kV/m for DC EF, 16.9 kV/m for AC EF, and 11.4 kV/m DC EF (with an additional 4 kV/m AC EF) in a hybrid condition. Neither ramp slope nor exposure duration influenced the participants' performance. Ion presence had no statistical significance on the participants’ sensitivity. Jankowiak et al. [13] observed an interaction between relative humidity and field type leading to the conclusion that high relative humidity (70%) facilitates DC EF perception, whereas low relative humidity (30%) supports the perception of AC EF. Despite the small number of participants, this study provided valuable insights into the feasibility of whole-body EF perception testing and served as a basis for parameter settings in this study, for example, by reducing ramp slope and exposure duration to a minimum of 3 and 5 s.

Referring to the International Commission on Non-Ionizing Radiation Protection (ICNIRP), a reference level of 5 kV/m was suggested for AC EF ranging from 25 to 300 Hz to limit the adverse effects on individuals [14]. Although a reference level of 20 kV/m was described in the frequency range of 1–8 Hz [14], no reference level for DC EF was provided [15]. The Institute of Electrical and Electronic Engineers (IEEE) recommends a maximum of 10 kV/m as the exposure reference level for DC EF [16]. Beyond these recommendations and although (a) HVDC overhead power lines are at work already in China, Canada, and the United States and (b) hybrid power lines are in the planning stage, the basis of the conscious perception of related EFs and the underlying physiological mechanisms are not completely understood. In addition, no recommendations or reliable findings on the human perception of hybrid EF exist, which renders research on this topic urgent and necessary [17–20].

### Aim

This research was motivated by the ongoing changes in energy transmission and aimed to determine the human detection thresholds for DC EF with and without ions, AC EF, and hybrid EF based on at least 200 participants equally distributed over sex and four age groups between 20 and 79 years. Furthermore, the influence of relative air humidity and physiological factors on EF perception were targeted in this investigation.

## Methods

### Participants

In this study, 203 healthy participants between the ages of 20 and 79 years were included. The recruitment of the participants for this monocentric study was carried out by postings, Internet advertisements, and announcements in regionally distributed newspapers. Males and females were equally distributed over the four age groups (Table 1).

**Table 1:** Study participants: Mean [standard deviation (SD)] age (years) in four age groups for male (M) and female (F) participants. n refers to the number of the participants included

Age group	20–34 years		35–49 years		50–64 years		65–79 years	
Sex	M	F	M	F	M	F	M	F
Mean (SD) age	25.44 (3.39)	24.96 (3.33)	41.84 (4.96)	42.83 (4.45)	57.39 (4.53)	54.84 (4.17)	70.16 (3.73)	70.16 (3.73)
n	25	26	25	24	28	25	25	25

Exclusion criteria were the presence of electronic implants, not removable piercings, self-reported electromagnetic hypersensitivity, dermatosis or neurological disorders, and claustrophobia. As part of the medical evaluation before study inclusion, a detailed medical anamnesis was conducted by a physician. In addition, vital signs, signs of infection, and skin and neurological abnormalities were examined. Female participants of childbearing potential underwent a urinary β-hCG test to rule out pregnancy. Before testing, all participants were briefed about benefits and risks and signed informed consent. Each participant obtained an expense allowance of 100 Euro. The study was approved by the Ethics Committee of the Medical Faculty of the RWTH Aachen (EK320/15) and complied with the Declaration of Helsinki.

### Exposure laboratory

**Fig. 1:**
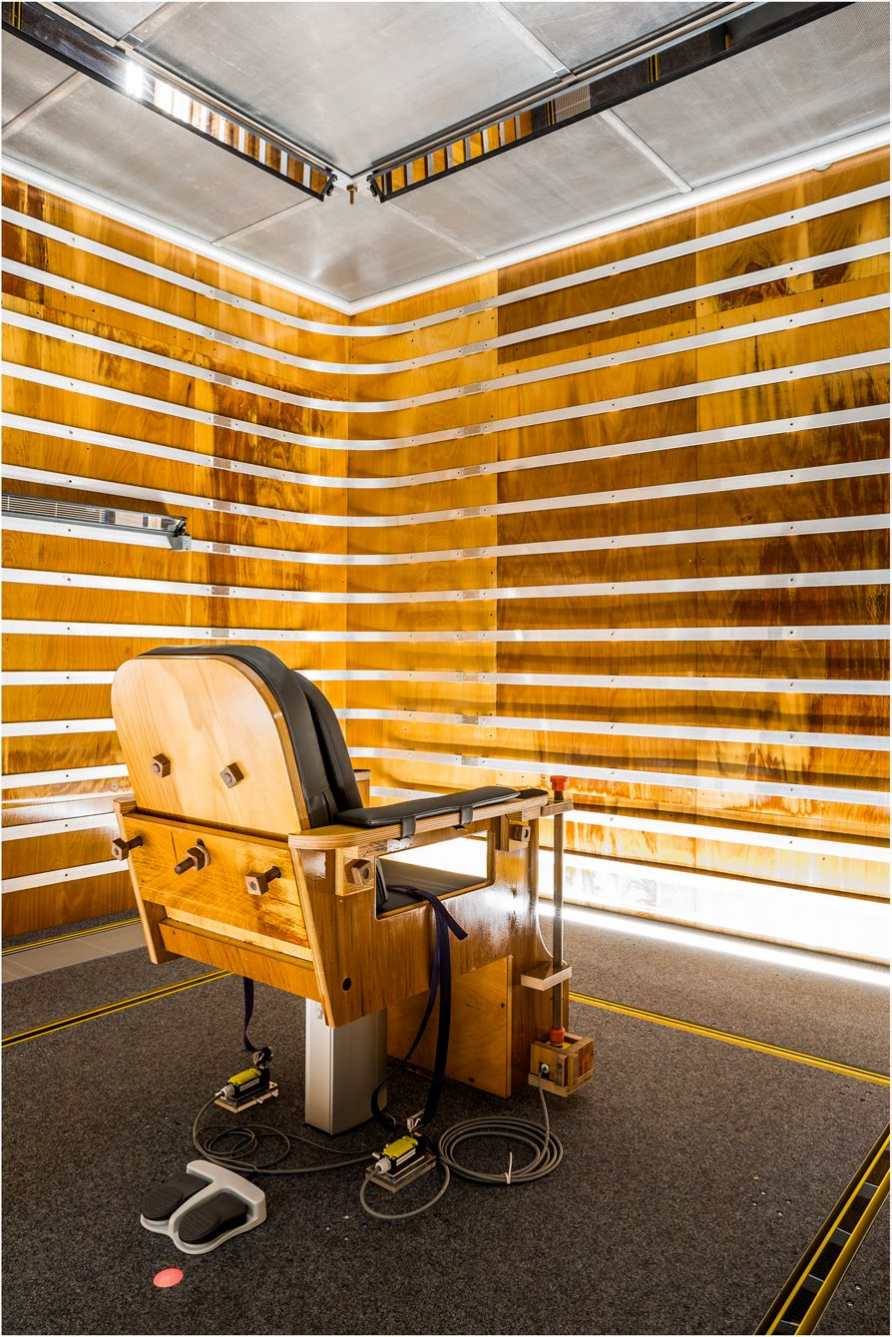
Photo of the exposure laboratory with the centralized chair. Photo made by Martin Braun Fotografie

A whole-body exposure laboratory located at the University Hospital RWTH Aachen was built in cooperation with the Institute for High Voltage Equipment and Grids, Digitalization and Power Economics, RWTH Aachen University. The setup of the exposure laboratory and the high-voltage system were similar to the laboratory described by Nguyen and Maruvada [12], which provided the basis of the experimental study of Blondin et al. [11]. A detailed description of the facility, including the high-voltage circuit and information on EF and ion generation as well as the measurement probes used, can be found in Jankowiak et al. [13]. In brief, the exposure laboratory had dimensions of 4 × 4 m and a height of 3 m (see Fig. 1). It was surrounded by a high-voltage technology area with an adjacent technical room, an investigator room, and an air-conditioning technology area. A height-adjustable wooden chair was located centrally in the exposure laboratory. At the right armrest, an optical response pad was affixed where the participants could insert their responses during experimental tasks. Several technical devices were located on ground level, such as a video projector displaying experimental information on the wall, an intercom for communication with the instructor, LED illumination, four loudspeakers, and two cameras. The walls of the exposure laboratory were built of laminated densified wood to provide good electric insulation, and the whole laboratory was placed on a sylomer layer to dampen external vibrations. Relative humidity within the exposure laboratory varied between 30 and 70%, and a temperature range between 20 °C and 27 °C was adjustable. The experimental sessions were conducted in a double-blind fashion, and possible auditory signals from the electrodes were masked by a 65.8 dB (A) white noise.

### High-voltage circuit

EFs and ion currents were generated via four 4 × 4 m electrodes stacked over each other at the ceiling of the exposure laboratory labeled as Electrode 1 (lowest), 2, 3, and 4. Electrodes 1, 2, and 4 were made of perforated aluminum sheets, whereas, within Electrode 2, 200 μm high-grade steel wires were mounted at a distance of 0.1 m. On the inside of the wooden walls, 14 grading electrodes were placed over each other and interconnected via ohmic-capacitive grading units. The floor of the exposure laboratory was used as a grounded base plate to which the participants were connected via silicone ankle bands (Schuler Medizintechnik, Freiburg, Germany) using a low resistive connection.

The four electrodes were connected to DC and AC voltage sources located in the high-voltage technology area. When energizing Electrode 1 by the combination of a 200 kV DC source and a 100 kV AC source, DC, AC, and hybrid EFs could be generated. The pair of Electrodes 2 and 4 as well as Electrode 3 were independently energized by two 20 kV DC sources. Applying voltage to Electrode 3 led to ion production, whereas the level of energizing Electrodes 2 and 4 determined the level of ion current flowing to the ground. With this arrangement, DC EF strengths of up to 50 kV/m and AC EF strengths with a maximum of 30 kV/m could be generated in the exposure laboratory. The superposition of both led to hybrid EF where a maximum of 50 kV/m (DC + AC_RMS_) was possible. Ion current densities could be generated with a maximum of 550 nA/m^2^ depending on the DC EF strength.

### Safety systems

To prevent the participants from approaching the high-voltage electrode system too closely, two redundant safety systems were integrated into the exposure laboratory. The participant’s chair was surrounded by light barriers (C4000, SICK, Waldkirch, Germany) providing an area of 2.08 × 2.08 m with a height of 1.67 m. Interrupting the light barriers led to an immediate shutdown of the high-voltage system in less than 180 ms. Additionally, a belt with contact plugs at its ends was used so that getting up from the chair also produced a shutdown of the high-voltage system. To provide a self-controlled action opportunity to the participants, an emergency switch was integrated at the end of the right armrest. Because ozone is a side product of corona discharge, a sensor of the model 106-L Ozone Monitor (2B Technologies, Kloten, Switzerland) was implemented to permanently measure accumulated ozone. To ensure the participant’s safety, two people were necessary during experimental testing: a technician was always present in the technical room confirming adequate working of the high-voltage system, while the investigator was present in the investigator room running experimental sessions and confirming that the participant appeared attentive at all times.

### EF values

In general, electric field strengths (*E*) can be reported in peak or rms values, where in DC EF *E*_RMS_ = *E*_Peak_ and in AC EF $\sqrt{2}$*E*_RMS_ = *E*_Peak_ for sinusoidal signals as applied here. For hybrid EFs this leads to ${E}_{\text{RMS}}=\sqrt{{{E}_{\text{DC}}}^{2}+{{E}_{\text{AC}}}^{2}}$ and ${E}_{\text{Peak}}={E}_{\text{DC}}+\sqrt{2}{E}_{\text{AC}}$ where *E*_AC_ is the rms value. AC_Peak_ values can be valuable for comparing the detection thresholds of AC and DC EFs [21]. By convention, unperturbed AC_RMS_ values are used e.g. by the ICNIRP or the IEEE suggesting reference levels [14, 16]. The same is true for previous studies on AC [7, 9] and hybrid EF perception [10]. Therefore, the current study used AC_RMS_ values to describe AC EFs. Moreover, AC EF, DC EF, and ions influence each other, which leads to complex compositions that affect the participants. The calibration process ensured that single EFs and ion current densities were homogenous in the unperturbed surrounding of the laboratory [13]. This includes assessing the EF values without ion enhancement.

### Psychophysiological methods

To measure the participant’s ability to perceive EF and calculate detection thresholds, two methods were used: signal detection theory (SDT; [22]) and a staircase procedure following the single interval adjustment matrix (SIAM; [23]). Derived from the SDT, the participant's task was to distinguish signal trials, where EF was present, from sham trials, where EF was absent. Therefore, a series of trials with varying EF strengths were randomly intermixed with the same number of sham trials. To take individual response bias into account, “yes” responses to signal trials (hit) and sham trials (false alarm) need to be considered. Thus, Green and Swets [22] suggested calculating d′ as an individual sensitivity index for a given signal strength by d′ = z (hit) – z(false alarm), where z refers to the standard score. Standard scores are calculated by z = (x – μ)/ σ, where x is the raw score, μ is the population mean, and σ is the population standard deviation. A sensitivity of 1 ≤ d′ < 2 is associated with successful signal detection; a sensitivity of 2 ≤ d′ < 3 reflects good sensitivity; and d′ ≥ 3 is associated with excellent sensitivity. The SIAM procedure is an adaptive algorithm following a participant’s responses to a given trial. Starting with a predefined EF, the strength within the following trial was reduced by 4 kV/m when the participant’s response was correctly “yes” or increased by 4 kV/m when the participant’s response was “no” referring to step size. After the false alarm, EF was increased by 8 kV/m. Correct responses to sham trials did not entail a change in EF strength. Reversals are defined as the point where the participant’s response leads to a direction change of the increasing or decreasing EF strength. After five reversals, the step size was reduced to 2 kV/m for a more fine-grained resolution of the last three reversals. The average of these three EF strengths was defined as an individual detection threshold that refers to the field strength where EF is just perceptible.

### Experimental procedure and parameter settings

Each participant attended the experimental testing on one day from 8 a.m. to 4 p.m. After medical review and assessment of physiological measures, the participants were carefully introduced to the technical components of the laboratory and all safety systems. Before starting with the experimental sessions, the participants were introduced to the expected perception characteristics described by the participants in the study of Jankowiak et al. [13], such as pleasurable tingling, slight itching, causes goosebumps, slight vibration, and formication. Additionally, the locations of the sensation were mentioned, such as scalp hair, face, and arms. Thereafter, the experimental task was described. The participant's task was to indicate via the response pad if they can perceive the presence of an EF. A trial started with a ramp duration of 3 s, where the EF increased from zero to the desired EF strength followed by a 5 s exposure duration. During the 4 s response period, where the EF was still present, a question was displayed via the video projector “Do you perceive an electric field?” Four buttons on the response pad were allocated to the responses “yes – certain,” “yes – uncertain,” “no – uncertain,” and “no – certain.” The decreasing period and intertrial interval lasted 7 to 9 s. The overall trial duration therefore was 20 s. Identical timing was used for sham trials, but no EF was present. The experimental task was carried out in 10 SDT sessions, each consisting of 40 trials with a duration of 15 min (in hybrid with ion sessions half of the trials), and 3 SIAM sessions with a variable duration between 5 and 20 min. Also, 15 min breaks after two or three sessions and a 1 h lunch break were included.

During the experimental tests, all participants wore long trousers and left their forearms uncovered. The temperature and relative humidity were set to 22 °C and 50%, respectively. Two SDT sessions for every EF type were carried out. The EF types were DC, DC with ion currents (DC_ION), AC, hybrid (H), and hybrid with ion currents (H_ION). In the DC_ION sessions, a specific ion current density with a positive polarity was assigned to a specific DC EF strength. This led to the combinations of 14 kV/m with 80 nA/m^2^, 22 kV/m with 200 nA/m^2^, 30 kV/m with 300 nA/m^2^, and 38 kV/m with 400 nA/m^2^. In hybrid EF sessions, 4 kV/m AC was always combined with various DC EF strengths, and an additional ion current density of 10 nA/m^2^ (H_ION) was provided. The proportion of sham trials was always 50%. For SIAM sessions, an initial EF strength was predefined that was 20 kV/m in DC and hybrid EFs and 14 kV/m in AC EF. Then, a corridor of EF strengths was provided for DC and hybrid EFs between 14 and 44 kV/m and for AC between 4 and 30 kV/m. Moreover, three termination criteria were necessary for SIAM sessions. When the participants reached three times the minimum or maximum EF strength of the corridor without (max) or with (min) successful detection, the session was terminated because the putative detection threshold was not within the provided EF strength corridor. When the participants showed a very liberal response behavior leading to many “yes” responses under uncertainty, the time until reversals were reached was long and thus exceeded the appropriate timescale. Therefore, the termination criterion “time” was applied after 20 min. The experimental parameters for SDT and SIAM sessions are depicted in Table 2.

**Table 2:** Experimental parameters of SDT and SIAM sessions: Every SDT session was conducted two times, except for the two subgroups where a lower or higher relative humidity was applied to half of the SDT sessions

Procedure	Label	DC EF strength (kV/m)	AC EF strength (kV/m)	Ion current densities (nA/m^2^)	n trials (sham trials)
SDT	DC	14, 22, 30, 38	-	-	40 (20)
	DC_ION	14, 22, 30, 38	-	80, 200, 300, 400	40 (20)
	AC	-	8, 16, 24, 30	-	40 (20)
	H	2, 8, 16, 24	4	-	40 (20)
	H_ION	18, 24	4	10	20 (10)
SIAM	DC	4–44; init. at 20	-	-	11–76 (50%)
	AC	-	4–30; init. at 14	-	8–68 (50%)
	H	4–44; init. at 20	4	-	11–70 (50%)

The participants in the two subgroups were exposed to half of the SDT sessions under 30% relative humidity (*n* = 24) or 70% relative humidity (*n* = 25). In both subgroups, the remaining SDT sessions were done under 50% relative humidity to allow intrasubject comparisons of the sensitivities. Age and sex were equally distributed across the subgroups, and the presentation order was counterbalanced across participants.

### Physiological measures

Because physiological characteristics at the body surface were shown to influence EF perception [9–11], skin moisture and vibrotactile perception were evaluated before starting EF perception testing. Skin moisture was measured at the inner part of the wrist on both arms and at the left neck below the jaw bone with a corneometer (CM 825; Courag + Khazaka electronic, Köln, Germany). Vibrotactile perception of the right index finger was measured using a perception meter (HVLab, Institute of Sound and Vibration Research, University of Southampton, Southampton, England). Five sessions at 16, 31, 63, 125, and 250 Hz were conducted each with a duration of 45 s. Within an increasing and decreasing amplitude, the participants had to continuously press a button when they perceived the vibration and had to release the button as soon as they perceive nothing. Vibrotactile detection thresholds in decibels were calculated by averaging reversals.

### Statistical analyses and data processing

When calculating sensitivity indices, z-transformation for hit and false alarm rates of 0 and 1 is not possible. Therefore, a log-linear correction according to Hautus [24] was done. Sensitivity indices were analyzed with SPSS 25 (IBM, Armonk, New York). Repeated-measures analyses of variances (rm ANOVA) were conducted on the dependent variable d′ with independent factors *EF strength* (DC: 14, 22, 30, and 38 kV/m; AC: 8, 16, 22, and 30 kV/m; hybrid: 2, 8, 16, and 24 kV/m; hybrid with ions: 18 and 24 kV/m) and *ion presence* (with ions and without ions). Examining relative humidity for each subgroup, rm ANOVAs with the additional independent factor *relative humidity* (30% and 50% or 70% and 50%) were conducted on sensitivity indices. An α-level of 5% was accepted for significance. When sphericity was violated, uncorrected degrees of freedom were presented together with corrected *F* and *p* values according to Greenhouse and Geisser [25]. For effect sizes, estimated partial η^2^ (η_p_^2^) were provided. To estimate detection thresholds, individual psychometric functions were calculated for each EF type. The association between physiological measures, age, sex, and EF perception ability was tested via correlational analyses between vibrotactile thresholds, skin moisture, age, sex and estimated detection thresholds. SDT detection thresholds were preferred because they were based on a larger number of participants compared to SIAM detection thresholds. The level of significance was Bonferroni corrected. Depending on the criterion for normal distribution statistically determined using the Kolmogorov–Smirnov test, Pearson’s *r* or Spearman’s ρ was presented. The last three reversals measured by the SIAM procedure were averaged to obtain individual detection thresholds, and data were discussed on a descriptive level.

## Results

### Effects of field type

**Fig. 2:**
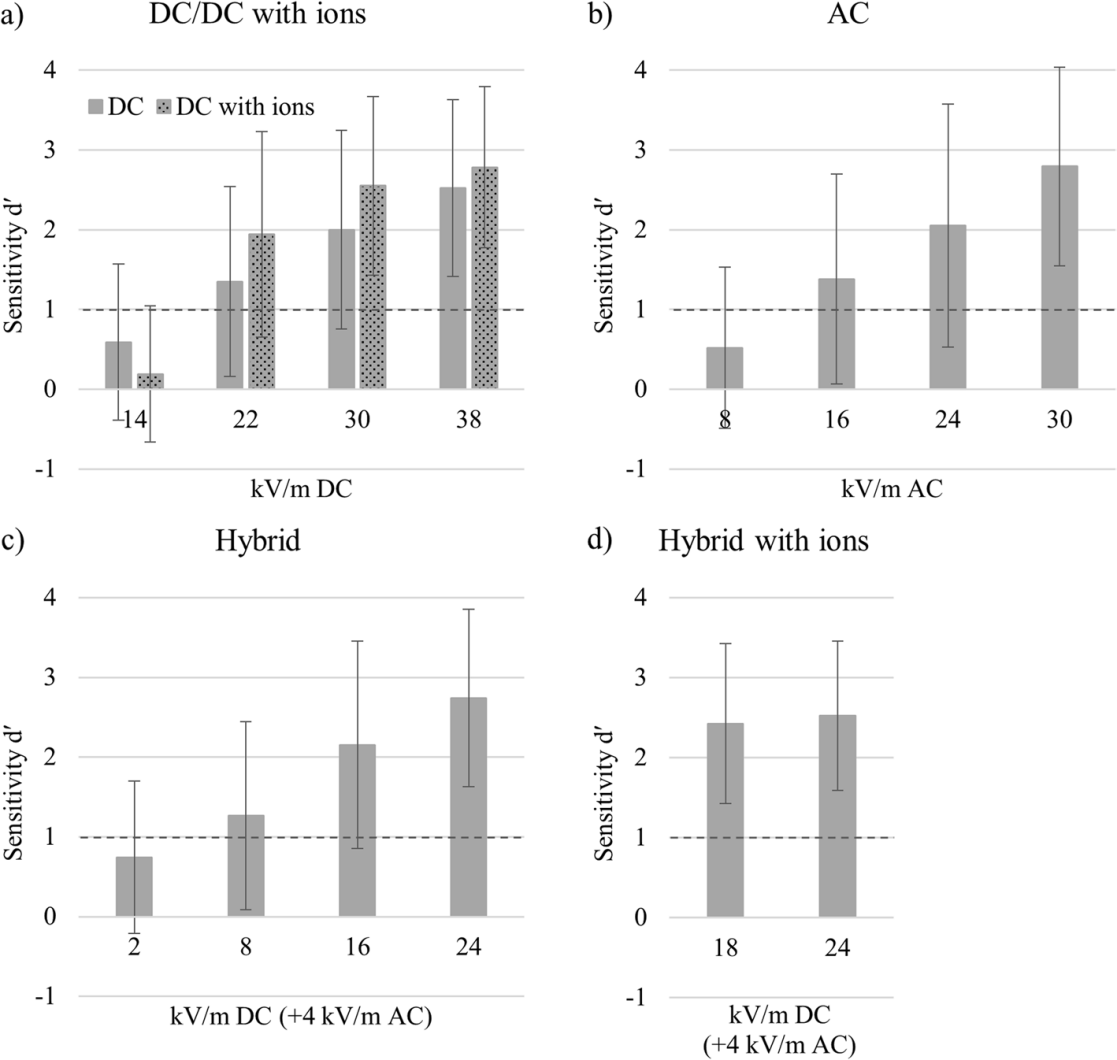
Sensitivity indices d′ are depicted for different EF types: **a** DC EF and DC EF with ions. Ion current densities were 80 nA/m^2^ at 14 kV/m, 200 nA/m^2^ at 22 kV/m, 300 nA/m^2^ at 30 kV/m, and 400 nA/m^2^ at 38 kV/m; **b** AC EF; **c** hybrid EF: various DC EF (x-axis) with a constant AC EF of 4 kV/m; and **d** hybrid EF with ions: various DC EF (x-axis) with a ion current density of 10 nA/m^2^ and a constant AC EF of 4 kV/m. Bars reflect SDs. n = 203 participants

*DC.* Sensitivity indices were statistically evaluated performing an rm ANOVA with *ion presence* (with ions and without ions) and *EF strength* (14, 22, 30, and 38 kV/m). A significant main effect of *EF strength* [*F* (3,199) = 70.04, *p* < 0.001, η_p_^2^ = 0.26] and an interaction of both factors [*F* (3,199) = 12.21, *p* < 0.001, η_p_^2^ = 0.06] were observed. This interaction effect represents the varying influence of ion presence on different EF strengths. Therefore, a paired sample t-test for *ion presence* was conducted for every level of *EF strength* (for 14 kV/m [*t* (202) = 5.07, *p* < 0.001], for 22 kV/m [*t*(202) =  − 6.25, *p* < 0.001], for 30 kV/m [*t* (202) =  − 6.22, *p* < 0.001], for 38 kV/m [*t*(202) =  − 3.56, *p* < 0.001]). As depicted in Fig. 2a, various d′ < 1 at 14 kV/m are responsible for the significant interaction effect between *field strength* and *ion presence*. However, these values are below the detection threshold. Thus, post hoc analysis were performed on EF strengths of average d′ > 1 using *EF strength* (22, 30, and 38 kV/m) and *ion presence* (with ions, without ions). Significant main effects for *EF strength* (*F*(2,200) = 25.56, *p* < 0.001, η_p_^2^ = 0.12) and *ion presence* (*F*(1,201) = 8.40, *p* < 0.005, η_p_^2^ = 0.04) as well as a marginally significant interaction between both factors (*p* = 0.06) were found.

*AC.* An rm ANOVA with the factor *EF strength* (8, 16, 24, and 30 kV/m) was conducted showing a significant main effect [*F*(3,199) = 38.65, *p* < 0.001, η_p_^2^ = 0.16]. In Fig. 2b, higher performance with increasing EF strength was observed.

*Hybrid.* To statistically examine the influence of different DC EF strengths under a constant AC EF of 4 kV/m on sensitivity indices, an rm ANOVA with the factor *EF strength* (2, 8, 16, and 24 kV/m) was performed. A significant main effect was found [*F*(3,199) = 21.28, *p* < 0.001, η_p_^2^ = 0.1]. When additional ions were presented in hybrid sessions, an rm ANOVA with factor *EF strength* (18 and 24 kV/m) did not show a significant effect (*p* > 0.59; see Fig. 2c and d).

As expected, the sensitivity index d′ rose with increasing EF strengths in all EF types, except for hybrid EF with ions. On average, the lowest EF strength investigated in each condition [DC (14 kV/m), DC with ions (14 kV/m), AC (8 kV/m), and hybrid EF (2 kV/m)] could not be detected successfully by the participants as indicated by a d′ < 1. Additionally, the presence of an ion current did influence the participants’ performance in DC EF conditions leading to an increased sensitivity especially when the EF strength was higher.

**Fig. 3:**
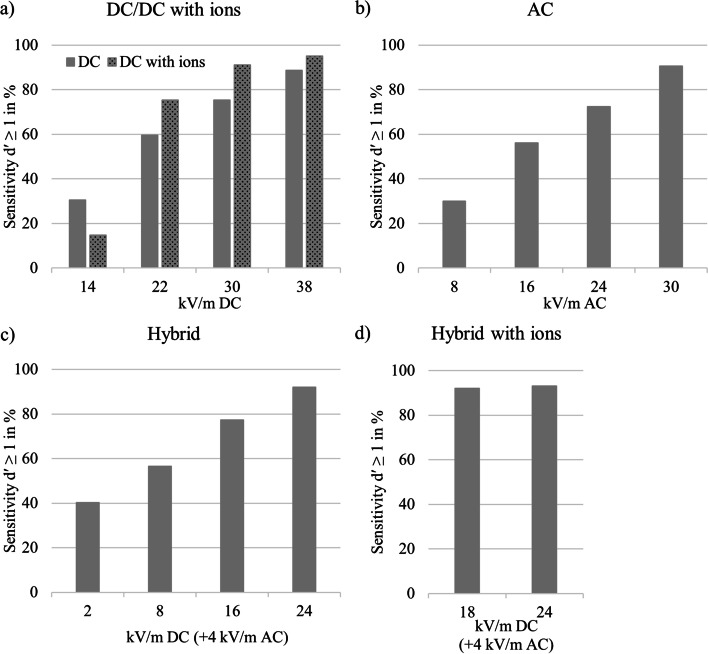
Percentage of participants (*n* = 203) showing sensitivity index d′ ≥ 1 for different EF types: **a** DC EF and DC EF with ions. Ion current densities were 80 nA/m^2^ at 14 kV/m, 200 nA/m^2^ at 22 kV/m, 300 nA/m^2^ at 30 kV/m, and 400 nA/m^2^ at 38 kV/m; **b** AC EF; **c** hybrid EF: various DC EF (x-axis) with a constant AC EF of 4 kV/m; and **d** hybrid EF: various DC EF (x-axis) combined with an ion current density of 10 nA/m^2^ and a constant AC EF of 4 kV/m

Although the average group performance in low EF strengths did not reach the level of successful detection (d′ < 1), individual performance differed. Between 15% (DC with ions) and 40% (hybrid) of the participants could detect the lowest EF strength, and a growing amount of successful detections was observed with increasing EF strength (Fig. 3).

### Effects of relative humidity

**Fig. 4:**
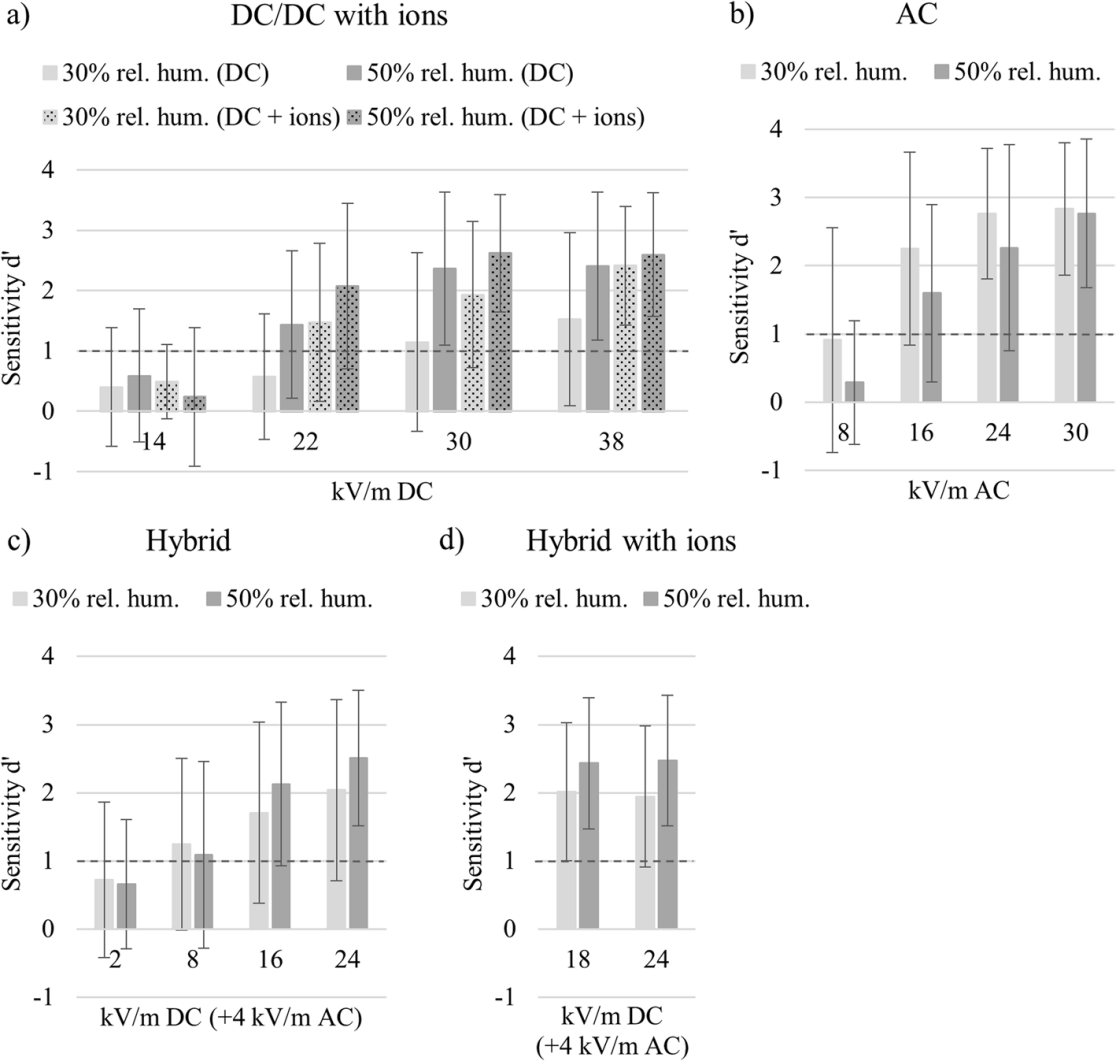
Sensitivity indices d′ under 30% and 50% relative humidity of a subgroup (*n* = 24): **a** DC EF and DC EF with ions. Ion current densities were 80 nA/m^2^ at 14 kV/m, 200 nA/m^2^ at 22 kV/m, 300 nA/m^2^ at 30 kV/m, and 400 nA/m^2^ at 38 kV/m; **b** AC EF; **c** hybrid EF: various DC EF (x-axis) with a constant AC EF of 4 kV/m; and **d** hybrid EF with ions: various DC EF (x-axis) combined with an ion current density of 10 nA/m^2^ and a constant AC EF of 4 kV/m. Bars reflect SDs

30% vs. 50% relative humidity. *DC.* An rm ANOVA with *relative humidity* (30% and 50%), *ion presence* (with ions and without ions), and *EF strength* (14, 22, 30, and 38 kV/m) yielded a significant main effect of *relative humidity* [*F*(1,23) = 17.79, *p* < 0.001, η_p_^2^ = 0.44] and an interaction of *relative humidity* and *EF strength* [*F*(3,21) = 9.55, *p* < 0.001, η_p_^2^ = 0.29]. In Fig. 4a, sensitivity indices were higher on average when the relative humidity was 50%.

*AC*. Analyzing sensitivities using an rm ANOVA with the factors *relative humidity* (30% and 50%) and *EF strength* (8, 16, 24, and 30 kV/m) showed a significant main effect of *relative humidity* [*F*(1,23) = 6.05, *p* < 0.05, η_p_^2^ = 0.21] but no interaction effect (*p* = 0.16). In contrast to the DC EF exposure, sensitivity was lower when the relative humidity was higher (50%, see Fig. 4b).

*Hybrid*. Conducting an rm ANOVA with the factors *relative humidity* (30% and 50%) and *EF strength* (2, 8, 16, and 24 kV/m) failed to reach significance (all *p's* > 0.06). When additional ions were presented, an rm ANOVA with the factors *relative humidity* (30% and 50%) and *EF strength* (18 and 24 kV/m) revealed a significant main effect for *relative humidity* [*F*(1,23) = 6.89, *p* < 0.05, η_p_^2^ = 0.23]. The participants’ performance increased when the relative humidity was high in hybrid sessions with ions, whereas, when no ions were presented, the factors *relative humidity* and *EF strength* only interacted on a descriptive level without reaching significance (see Fig. 4c and d).

**Fig. 5:**
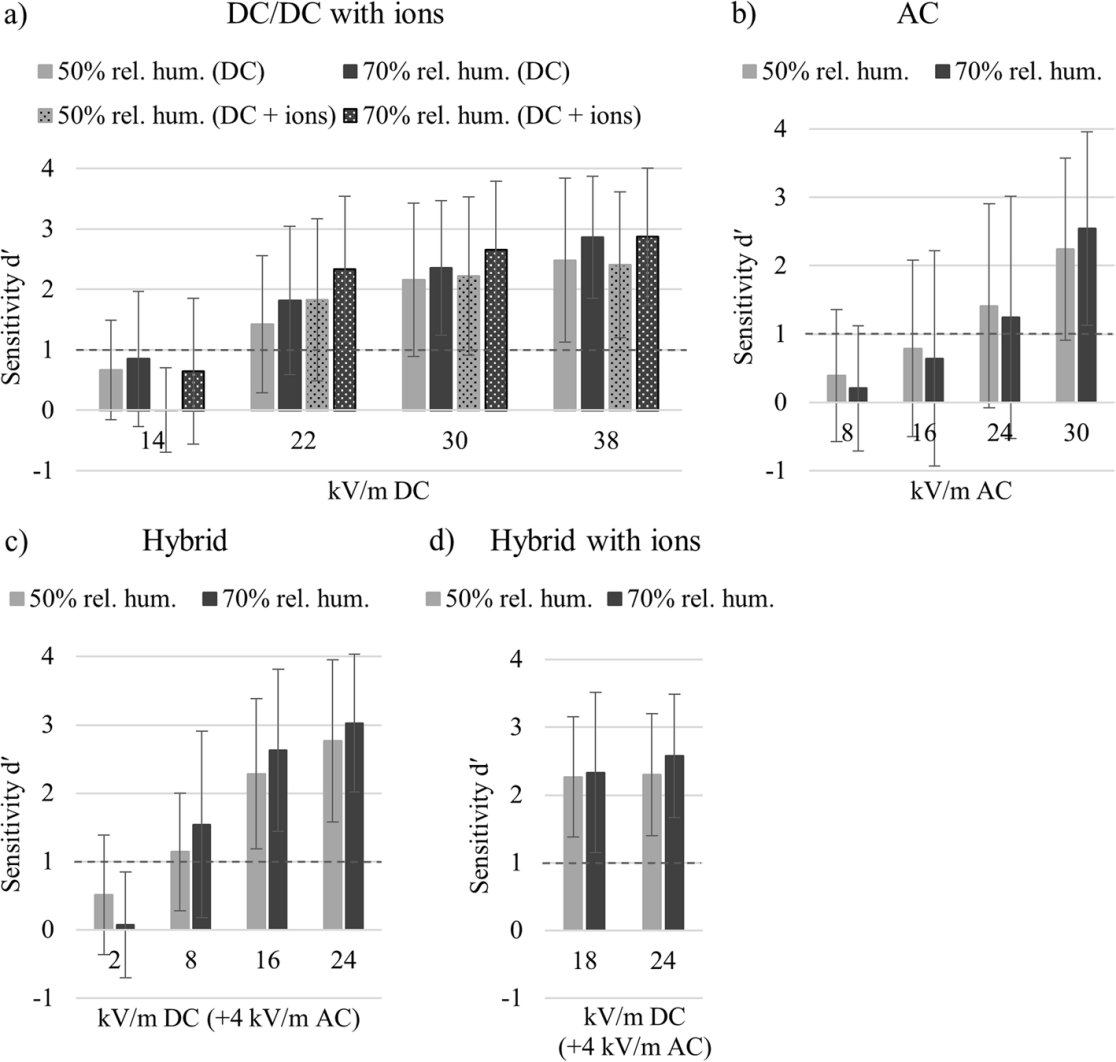
Sensitivity indices d′ under 50% and 70% relative humidity of a subgroup (*n* = 25): **a** DC EF and DC EF with ions. Ion current densities were 80 nA/m^2^ at 14 kV/m, 200 nA/m^2^ at 22 kV/m, 300 nA/m^2^ at 30 kV/m, and 400 nA/m^2^ at 38 kV/m; **b** AC EF; **c** hybrid EF: various DC EF (x-axis) with a constant AC EF of 4 kV/m; and **d** hybrid EF with ions: various DC EF (x-axis) combined with an ion current density of 10 nA/m^2^ and a constant AC EF of 4 kV/m. Bars reflect SDs

50% vs. 70% relative humidity. *DC*. To statistically evaluate how the modification of relative humidity from 50 to 70% influences sensitivity indices, an rm ANOVA with the factors *relative humidity* (50% and 70%), *ion presence* (with ions and without ions), and *EF strength* (14, 22, 30, and 38 kV/m) was performed. Only a significant main effect of *relative humidity* was found [*F*(1,24) = 16.55, *p* < 0.001, η_p_^2^ = 0.41]. No other effects, including the factor *relative humidity*, were observed (all *p's* > 0.31). In Fig. 5a, general performance was higher when the relative humidity was set to 70% in comparison to 50% relative humidity.

*AC*. An rm ANOVA with the factors *relative humidity* (50% and 70%) and *EF strength* (8, 16, 22, and 30 kV/m) did not show any significant effects, including *relative humidity* (all *p* > 0.31; see Fig. 5b).

*Hybrid*. Performing an rm ANOVA with the factors *relative humidity* (50% and 70%) and *EF strength* (2, 8, 16, and 24 kV/m) revealed a significant interaction effect of both factors [*F*(3,22) = 5.44, *p* < 0.01, η_p_^2^ = 0.19]. Analyzing sensitivity indices in hybrid sessions with ions, no significant effects were found (all *p's* > 0.18). When presenting 8, 16, and 24 kV/m, the participants’ performance was increased with 70% relative humidity (see Fig. 5c).

### Detection thresholds

Exposure to the predefined EF types and levels in SDT sessions enabled the calculation of individual psychometric functions using linear interpolation. Therefore, two preconditions were necessary: the participants were required to reach at least d′ ≥ 1 for one EF strength per EF type, and the distribution of sensitivity indices over all EF strengths should be plausible. Plausibility is defined as performance must not drop below the critical value of d′ = 1 for a given EF strength, when in lower EF strengths a d′ ≥ 1 was observed. For example, the participants were excluded when they yielded a d′ ≥ 1 at 14 kV/m but a d′ < 1 at 22 kV/m. Thus, 7% to 19% of the participants were excluded in each field type. The detection thresholds referring to the calculated EF strength where d′ = 1 is reached are illustrated in Table 3.

**Table 3:** SDT and SIAM detection thresholds. SDT: Mean values of the calculated detection thresholds derived from individual psychometric functions for DC EF (*n* = 165), DC EF with ions (*n* = 189), AC EF (*n* = 175), and hybrid EF (*n* = 168). SIAM: Detection thresholds were calculated from the last three reversals for DC EF (*n* = 134), AC EF (*n* = 134), and hybrid EF (*n* = 124). SDs are enclosed in parentheses

Method	DC	DC with ions	AC	Hybrid
SDT (kV/m)	18.69 (8.42)	18.22 (5.65)	14.16 (7.96)	6.76 (6.26) DC + 4 AC
SIAM (kV/m)	23.17 (8.98)		17.57 (7.55)	14.34 (6.29) DC + 4 AC

**Fig. 6:**
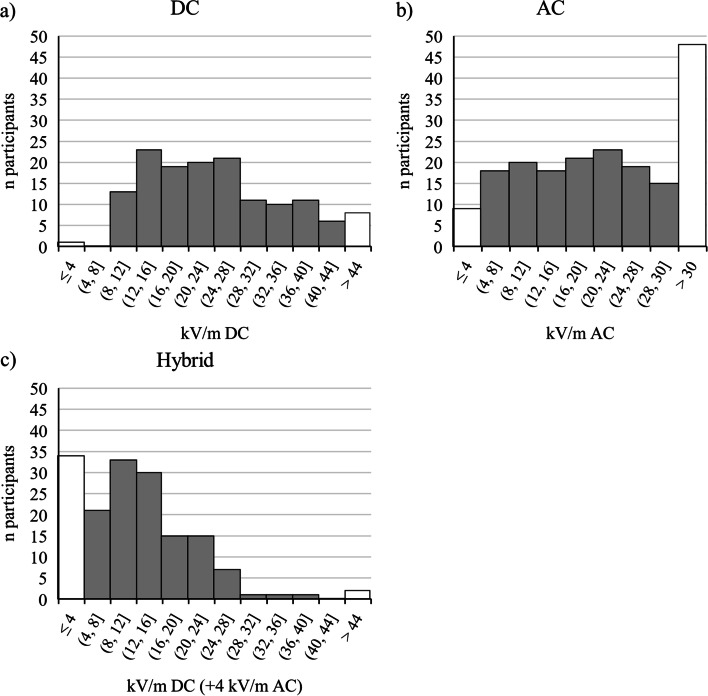
Histograms of SIAM detection thresholds: For **a** DC EF, **b** AC EF, and **c** hybrid EF. In hybrid sessions, various DC EFs (x-axis) were always combined with an AC EF of 4 kV/m. White bars at the left and right edges reflect the participants with min or max termination criteria, where no detection threshold could be assessed

Table 3 presents the SIAM detection thresholds. As shown in Fig. 6a and c, the majority of detection thresholds for hybrid EF was found to be within the lower half of the given range, which is in contrast to those of the DC detection thresholds. In AC EF, 48 participants were unable to detect the maximum EF strength of 30 kV/m (max termination criterion; Fig. 6b).

### Effects of physiological measures, age, and sex

Correlational matrices between EF detection thresholds (DC EF, DC EF with ions, AC EF, and hybrid EF) and measures of skin moisture (left and right arm and left neck) were not significant. In contrast, data analyses of vibrotactile measures showed a significant correlation of AC EF detection threshold and vibrotactile measures at 31 Hz (ρ = 0.25, *p* = 0.001) and hybrid EF detection threshold and vibrotactile measures at 31 Hz (ρ = 0.25, *p* = 0.001) and 63 Hz (ρ = 0.21, *p* = 0.007). Thus, higher EF detection thresholds were associated with poor sensitivity toward vibrotactile assessment. After Bonferroni correction, none of the correlational analyses between detection thresholds and age remained significant (all *p's* > 0.015). Correlational analyses between sex and detection thresholds showed a significant correlation between sex and DC with ions (*r* =  − 0.20, *p* = 0.007). Although a trend toward lower detection thresholds among the female participants was observed compared with the male participants, no other correlational analyses yielded significance (all *p'*s > 0.03).

## Discussion

This research project aimed to examine human detection thresholds for different EFs in view of the development in energy transmission. To the authors’ knowledge, this is the first investigation of hybrid EF perception in a whole-body setup under highly controlled conditions. Therefore, 203 participants were exposed to various EFs with experimental parameters assessed in a prestudy [13]. Detection thresholds in hybrid EF were lower compared to DC and AC EF alone, and additional presentation of ions facilitated the perception of DC EF. Changes in the relative humidity in two subgroups altered the detection of DC and AC EFs in different ways. Correlational analyses of the physiological assessment showed an unspecific relation between vibrotactile measure and EF perception.

In general, the participant's ability to detect EF was better than the results from previous studies. Clairmont et al. [10] estimated perceptible DC EF at about 20 kV/m. Compared to Blondin et al. [11], who determined detection thresholds of 45.1 kV/m without ions and 36.9 kV/m with ions, the detection thresholds in this study were lower by half with 18.69 kV/m without ions and 18.22 kV/m with ions. Besides the four times higher number of participants, the relative humidity was set constantly to 50%. Blondin et al. [11] reported a relative humidity ranging between 6 and 40%. The subgroup analyses indicated that the low relative humidity reduced the participants’ ability to detect DC EF. When the relative humidity was 50%, sensitivity at 22 kV/m was d′ > 1. In contrast, when the relative humidity was 30%, the sensitivity was d′ > 1 only at 30 kV/m (see Fig. 4a). Thus, reduced relative humidity was associated with worse performance in DC EF, which could be a major reason for the differences between the results from Blondin et al. [11] and this study. In addition, Odagiri-Shimzu and Shimzu [8] provided support for this explanation, as they found high detection thresholds when relative humidity was set to 50% instead of 90%.

In this study, the average detection thresholds for DC EF without ions (18.69 kV/m) and with ions (18.22 kV/m) were very close. In terms of sensitivities, interpreting the interaction between *ion presence* and *EF strength* is difficult. Better performance was observed at 22, 30, and 38 kV/m in the presence of ions. Thus, the numerically reversed pattern at 14 kV/m cannot be interpreted as average sensitivities for both conditions were less than the critical value of 1. Regarding the SDT results of Blondin et al. [11], no significant differences between no-ion condition and intermediate-ion condition (60 nA/m^2^) were observed, whereas, in the high-ion condition, the performance significantly increased. In this study, an ion current density of 80 nA/m^2^ was used at 14 kV/m that may have led to a lack of a positive effect of ions on performance. When DC EF and ion concentration were higher, sensitivity increased significantly compared to DC EF without ions, which is in line with the results of Blondin et al. [11].

In hybrid EF, the detection threshold was 6.76 kV/m DC when 4 kV/m AC was additionally present. Clairmont et al. [10] observed a DC EF of 5 kV/m as just perceptible under coexposure of 10 kV/m AC EF. Compared to DC EF exposure alone, the combination of DC and AC EF seems to have a facilitating synergistic effect on human perception. As previous studies illustrated that body hair can enhance AC and DC EF perception [7, 8], the synergistic effect of both on EF perception may by partially evoked by intensified hair movements. Surprisingly, sensitivity indices indicated that 40% of the participants were able to successfully detect the minimum hybrid EF combination of 2 kV/m DC and 4 kV/m AC (see Fig. 3c). This outcome was not expected regarding the prestudy [13], where a hybrid EF threshold of 11.4 kV/m (SD = 2.9 kV/m) was observed, nor the results from Clairmont et al. [10] but could reinforce even more the synergistic effect of both EF types on human perception.

Because two approaches for estimating human perception of EF were used, the results differed at least when comparing the detection thresholds calculated from the SDT and SIAM procedures. SIAM detection thresholds were higher with 23.17, 17.57, and 14.34 kV/m for DC, AC, and hybrid EFs, respectively, compared to SDT thresholds with 18.69 kV/m (DC EF), 14.16 kV/m (AC EF), and 6.76 kV/m (hybrid EF). SIAM thresholds were based on a reduced number of participants as seen in Fig. 6. Thus, participants with min, max, or time termination criteria were not included, which may have biased the results. Especially in hybrid conditions, 34 participants were not included in SIAM thresholds because they could reliably perceive the lowest EF strength, which was likely to increase the average hybrid EF threshold. Nevertheless, the main finding showing a reduced detection threshold in hybrid EF compared to DC and AC EF alone was observed in both approaches. Lower detection thresholds in SDT compared to SIAM procedures were also found by Blondin et al. [11].

Changes in relative humidity showed an impact on the participants’ performance, which is in line with the results of the prestudy [13]. From the results, AC EF was easier to perceive when environmental air becomes dryer. In contrast, when the relative humidity was high, DC EF was easier to detect. In hybrid EF, the direction of this effect depended on the ratio of AC and DC. A higher amount of DC EF produced a result pattern typically observed in DC EF, i.e., the perception was facilitated by increased relative humidity. Increased DC EF perception when the relative humidity was high, is supported by findings of Odagiri-Shimizu and Shimizu [8] showing increased sensitivity of the forearm region to DC EF when the relative humidity was set to 90%. The observation of relatively good AC EF perception ability in low relative humidity contrasts the findings of Kato et al. [9], showing a reduced ability of AC EF perception in low relative humidity. However, the authors exposed only hand and forearm to AC EF of up to 115 kV/m. Focusing on the whole body could enable different mechanisms that contribute to EF perception. As Reilly [6] outlined, two mechanisms may be involved in AC EF perception: dry air may facilitate mechanical force on hair itself, whereas humid conditions may promote conductive characteristics of hair leading to mutual repulsion between hair follicles. Based on the data, we propose to expand this model by suggesting that the mechanical force impact under dry conditions is the more prominent perception mechanism in AC EF, whereas conductive processes under high humidity conditions are the main mechanism facilitating DC EF perception.

On a group level, skin moisture did not correlate with EF detection thresholds. Skin moisture was measured on the left and right arms above the wrist and on the neck at one time point before the start of the first session. Increased measurements during the day at different locations, which are more involved in EF perception, may have led to a significant effect. However, vibrotactile measures at 31 and 63 Hz revealed a correlation with AC and hybrid EF detection thresholds, respectively. For AC EF perception, it is plausible that good individual ability of vibration detection in the range of 50 Hz is associated with a good EF detection. Although a correlation of vibrotactile measures at 31 Hz and AC EF detection was found, this was not the case at 63 Hz. The link between hybrid EF perception and the ability to detect 31 and 63 Hz vibration frequency could be explained by the kind of sensation (tingling, itching, and vibration) that was described by participants earlier [13]. Moreover, previous studies demonstrated that sensitivity to oscillatory stimuli via body hairs is linked to the human sense of vibration [26]. In conclusion, the results indicate that sensibility at the body surface seems to be an important factor in the interaction mechanism of EF perception. Although no correlational link between age and perception ability was found, the female participants exhibited lower detection thresholds in DC EF with ions compared to those of the male participants. No general pattern at a significant level could be detected. However, future studies on EF perception should consider the trend of the influence of gender on EF perception.

On average, the SDT detection thresholds exceeded the reference level of 5 kV/m for AC EF provided by the ICNIRP [14] at 14.16 kV/m. However, in the SIAM sessions, 18 participants exhibited detection thresholds ranging from 4 – 8 kV/m (Fig. 6b). Moreover, the average SDT detection threshold in the current study of 18.69 kV/m for DC EFs is above the recommended reference level of 10 kV/m by the IEEE [16]. Nevertheless in the range of 8 – 12 kV/m detection thresholds of 13 participants were found in DC EF SIAM sessions (Fig. 6a). Since effects of ion current presentation were found in higher EF strengths (i.e., 22, 30, and 38 kV/m), calculated SDT detection thresholds with and without ions were very close.

### Limitations

Although data from all participants were included in sensitivity indices d′, detection thresholds were calculated based on a reduced set of participants. One requirement was that, at least in one EF strength, a d′ ≥ 1 was observed. Therefore, the participants with a detection threshold that was presumably above the maximum EF strengths were excluded. This may imply that the calculated detection thresholds were somewhat lower because only the participants who could detect at least one EF strength were included. The same argumentation should be made for SIAM thresholds, which as well are based on a reduced number of participants.

## Conclusion

This study is the first systematic investigation of hybrid EF perception in humans using a double-blind experimental setup. While detection thresholds of DC and AC EF were lower compared to previous studies, the synergistic effect of both field types on human perception was shown. Especially, in the minimal hybrid EF combination (2 kV/m DC and 4 kV/m AC), 40% of the participants were able to successfully detect the EF. In terms of unwanted sensory perception, this should be considered in the discussion on reference levels and recommendations for hybrid EFs, which current remain non-existent. Furthermore, we suggest a further investigation on low hybrid EFs in detail to obtain a fine-grained picture of the lower bound of human EF perception. Although the average detection thresholds do not undercut the existing reference levels for DC and AC EFs, the study found evidence for successful EF perception around these reference levels in a small subset of participants. As the reference levels were mainly designed to prevent adverse health effects during EF exposure [14, 16], the current results do not question these values. Furthermore, the findings can be utilized in the discussion on whether sensory perception, as a byproduct of EF exposure, is acceptable.

We enhanced the external validity of the results by investigating subgroups across different levels of relative humidity. Hints for an enhanced perception of DC EF in a high-humidity environment and a facilitated AC perception under low-humidity conditions were confirmed. These results may further contribute to the understanding of the underlying mechanisms and should be considered as well in the discussion on recommendations for EF exposure. Thus, this study provided reliable EF detection thresholds that add quantitative information on sensory perception to the discourse on the impact of EF exposure. In this manner, it can help to improve the construction processes of planned HVDC and hybrid overhead power lines.

## Data Availability

The datasets used and/or analysed during the current study are available from the corresponding author on reasonable request.

## Abbreviations

AC: Alternating current
dB (A): Decibels (A)
DC: Direct current
*E*: Electric field strength
EF: Electric field
h: Hour
HVAC: High-voltage alternating current
HVDC: High-voltage direct current
Hz: Hertz
kV/m: Kilovolt/meter
nA/m^2^: Nanoampere per squaremeter
rm ANOVA: Repeated measures analyses of variance
s: Second
SD: Standard deviation
SDT: Signal detection theory
SIAM: Single interval adjustment matrix
